# Interactions Between Genes From Aging Pathways Significantly Influence Risk of Alzheimer’s Disease

**DOI:** 10.1093/geroni/igaa057.467

**Published:** 2020-12-16

**Authors:** Svetlana Ukraintseva, Konstantin Arbeev, Hongzhe Duan, Igor Akushevich, Mary Feitosa, Kaare Christensen, Eric Stallard, Anatoliy Yashin

**Affiliations:** 1 Duke University, Durham, North Carolina, United States; 2 Washington University School of Medicine, St Louis, Missouri, United States; 3 University of Southern Denmark, Odense C, Syddanmark, Denmark; 4 Duke University, Biodemography of Aging Research Unit, Durham, North Carolina, United States

## Abstract

Age is major risk factor for AD; however, relationships between aging and AD are not well understood. Decline in physiological resilience is universal feature of human aging that may also play role in AD. Aging-related pathways (such as IGF-I/P53/mTOR-mediated) that are involved in tissue resilience work in concert to decide outcomes of cell responses to stress/damage, such as survival, apoptosis, autophagy, etc. We hypothesized that interplay among genes in these pathways may influence AD risk as result of epistasis (GxG). We estimated effects of pairwise epistasis between SNPs in 53 genes from respective pathways on AD risk in the LLFS compared with other data (HRS, CHS, LOADFS). We found significant (fdr<0.05) GxG effects on AD risk in older adults across datasets. The SNP rs11765954 in CDK6 gene was involved in top GxG effects on AD in all datasets, when paired with SNPs in BCL2 and PPARGC1A. The CDK6 role in AD could be pleiotropic, depending on its activity in neurons: CDK6 expression is needed for DNA repair and neuronal survival; however, CDK6 overexpression may lead to the cell cycle reentry in postmitotic neurons resulting in apoptosis, which may contribute to neurodegeneration. CDK6 was earlier found to interfere with BCL2 effects on apoptosis, and with PPARGC1A effects on energy metabolism, which might contribute to observed GxG between these genes. We conclude that interactions among genes from biologically connected aging pathways may significantly influence AD risk. Uncovering such GxG effects has a potential to yield new genetic targets for AD prevention/treatment.

